# Facts in Homœopathic Prescribing

**Published:** 1894-08

**Authors:** 


### HOMŒOPATHIC PHYSICIAN,

A MONTHLY JOURNAL OF

HOMŒOPATHIC MATERIA MEDICA AND CLINICAL MEDICINE.

" If our school ever gives up the strict inductive method of Hahnemann, we are lost, and deserve only to be mentioned as a caricature in the history of medicine."—CONSTANTINE HERING.

Vol. XIV. AUGUST, 1894. No. 8.

### EDITORIAL.

FACTS IN HOMŒOPATHIC PRESCRIBING.—When a physician prescribes rationally upon a pathological basis he is really prescribing upon speculative views.

Filled with a vivid mental picture of the pathology of the case, the image in his mind is so attractive that he is *sure* he is right, and when he reasons out the course of treatment he is so well satisfied that he prescribes massive doses of drugs without the slightest hesitation.

Thus he is blinded by his theoretical images so completely that he scarcely realizes any deleterious effect of his medicines.

No misgiving does he have that his view is erroneous. Yet the history of medicine shows that the time will arrive when he will surely discard the theory which he has trusted so implicitly and set up another in its place.

Certainly we will not be surprised that much error must prevail, much damage be done, and much failure be experienced when we reflect that so much of medical treatment depends upon untrustworthy *views* of the nature of the diseased state, and upon deductions as to treatment which by reason of being dependent upon these views must be still more unreliable and even dangerous.

If the pathologist be governed by facts, he, more than the homœopathist, deserves the term which he has made invidious

—"Symptom Coverer." If the patient have a high fever, the pathologist does not hesitate to immerse the sufferer's whole body in a bath of ice-cold water, and that, too, when dealing with such fell diseases as pneumonia and typhoid fever. If the bowel be sluggish, it is urged by a purgative. If it be too lax, an agent that will practically plug it up is administered, and so on through the list. Remedies thus given for individual symptoms are selected without any regard to their influence upon the other symptoms in the case, and as each symptom thus has its own therapeutic measure, a wondrous structure of poly-pharmacy is reared, where the drugs are combined, not with regard to their respective pathogenetic effects, but with reference to their chemical reaction upon each other.

The view here given does not, we believe, do injustice to the dominant school.

In contrast with it we have the position occupied by our own school.

The formula *Similia Similibus Curantur*, under which we prescribe, is the expression of a natural *law*. Our very first step, therefore, is a certainty. Then we have a materia medica composed of provings of drugs upon the healthy. This is a catalogue of pathogenetic *facts*. The list of symptoms given by the patient and carefully written down by the physician constitutes another set of facts.

The remedy being selected because the pathogenetic facts produced by it correspond with the symptomatic facts of the patient's condition, in obedience to the *law* of the Similars, puts the prescriber at once above the plane of speculation and upon the more "solid ground of nature."

Therefore in such a scheme there is no room for any follies of diagnosis, which are after all only speculative views. There can be no therapeutic measures based upon opinion. There can be no jeopardizing of the patient's chances of recovery by reason of undiscovered errors in these pathological reasonings, and there can be no chagrin or self-reproach for the lamentable consequences of such errors.

The homœopathist who is true to his professions has, therefore, a pearl of great price in this method of dealing with sickness.

Do the homœopathists as a body realize the value of this possession of theirs? We think not. They can hardly be appreciative of it, else they would not imitate the old-school pathology. They would not prescribe, as do the members of the old school, massive doses of drugs on pathological conclusions. They would not show the contempt they do for strict homœopathists, nor so persistently ignore their teachings and example.

The editor had a recent experience with one of these "liberal" homœopathists which has incited him to the foregoing reflections, and to the publication of them, in the hope of influencing some readers of the journal who are too much addicted, in the treatment of their cases, to contemplating the "pathological picture-book" as it was called by the late Dr. Lippe.

The experience referred to was a case of Bright's disease abandoned by the "liberal" as hopeless and evidently within a few hours of death. A well-known New York physican was then called in, who gave the truly indicted simillimum which at once made a favorable change in the patient and gave promise of recovery. The case was then turned over to the writer, who continued the same treatment as his colleague.

The recovery of the patient was as miraculous as it was gratifying. The "liberal," when he beheld the change in the patient's condition, declared, in the face of the fact of the improvement, that there was no medicine at all in the pills prescribed by his successor, but that he, the "liberal," was giving "something to heal the kidneys." This "something," it may be incidentally mentioned, was whiskey and water!

Certainly it would seem that a physician who places himself in such a position as is here shown stands much in need of these remarks. Yet it is equally certain he is so sure of his philosophy that notwithstanding the fact of the result, he is still unimpressed, still untroubled by any impulses to an examination of his method, still incapable of learning the lessson.

In the words of Holy Writ: "If they hear not Moses and the prophets, neither will they be persuaded though one rose from the dead."

